# Measuring Consumer Engagement in Omnichannel Retailing: The Mobile In-Store Experience (MIX) Index

**DOI:** 10.3389/fpsyg.2021.661503

**Published:** 2021-04-13

**Authors:** Charles Aaron Lawry, Anita D. Bhappu

**Affiliations:** ^1^Division of Consumer Science, College of Health and Human Sciences, Purdue University, West Lafayette, IN, United States; ^2^Gallo Management Program, School of Engineering, University of California, Merced, Merced, CA, United States

**Keywords:** m-commerce, mobile marketing, retail services, retail technology, formative measures, activity theory, fashion retailing, consumer behavior

## Abstract

We draw insights from Activity Theory within the field of human-computer interaction to quantitatively measure a mobile in-store experience (MIX), which includes the suite of shopping activities and retail services that a consumer can engage in when using their mobile device in brick-and-mortar stores. We developed and validated a nine-item, formative MIX index using survey data collected from fashion consumers in the United States (*n* = 1,267), United Kingdom (*n* = 370), Germany (*n* = 362), and France (*n* = 219). As survey measures of consumer engagement in omnichannel retailing using a mobile device, the index items with stronger factor loadings described in-store shopping activities whereas those with weaker factor loadings described activities related to behavioral targeting and social networking. These results suggest that retailers should give consumers the autonomy to independently find, evaluate and purchase merchandise in brick-and-mortar stores, thereby enabling them to co-create personalized shopping experiences as active participants within an omnichannel retail servicescape. Our findings also suggest that retailers should provide consumers with more authentic ways to build community and brand affiliations than mobile marketing and social media promotions. In-store activities should not simply be a migration of pre-existing e-commerce capabilities onto mobile devices. An engaging mobile in-store experience should be an amalgam of physical and digital activities that produce a seamless shopping journey and leverage the unique properties of mobile devices – ultra-portability, location sensitivity, untetheredness, and personalization. Retail executives can use the validated MIX index to prepare strategic investments in mobile technology applications and capabilities for retail stores within their omnichannel operations. The nine-item MIX index is also well-suited for consumer surveys, which also makes it an attractive measure of consumer engagement in omnichannel retailing for future academic research.

## Introduction

In retailing today, consumers expect a seamless shopping experience across different retail channels (Blázquez, 2014; Lemon and Verhoef, 2016). One way that retailers can deliver this omnichannel experience is to engage consumers with mobile devices in activities that connect them to retailers' digital commerce platforms, especially while consumers are shopping in their brick-and-mortar stores (Saarijärvi et al., 2014; Hatter, 2015). It is estimated that nearly 78% of young adults use their mobile devices while shopping in-store (Briggs, 2019). In fact, 46% of all digital retail orders and 65% of all traffic to retail websites during the first quarter of 2019 were generated from a mobile device (Salesforce Commerce Cloud, 2019; Charlton, 2020), which represented a new record for global m-commerce. These robust figures suggest that retailers need to understand their target customers' preferences for using a mobile device when shopping in order to make the best strategic investments in mobile services and mobile marketing campaigns.

Mobile devices can influence all stages of the shopping journey, including purchase planning and purchase execution at home or in-store, thereby enabling an omnichannel customer experience with no defined starting or ending point (Payne et al., 2017; Sopadjieva et al., 2017). Most retailers are, therefore, adopting a digital commerce platform that “manages all consumer interactions and transactions throughout the consumer shopping journey” (National Retail Federation, 2015, p. 2). By evolving their omnichannel capabilities through mobile in-store services, retailers can leverage their existing product, order, and customer data to enhance the overall shopping journey (National Retail Federation, 2014). Even so, research on how to effectively measure and increase mobile shopping engagement is still lacking (Shankar et al., 2016). The retail innovations and services literature has qualitatively described some activities that may comprise in-store mobile adoption and use (Bézes, 2019; Mishra et al., 2021; Alexander and Kent, in press). However, no quantitative studies to date have tested consumers' assessment of a mobile in-store experience, which includes the suite of shopping activities and retail services that a consumer can engage with when using their mobile device in brick-and-mortar stores.

To facilitate academic research and strategic investment in omnichannel retailing, we draw insights from Activity Theory (AT) within the field of human-computer interaction to quantitatively measure a mobile in-store experience (MIX), which includes the suite of shopping activities and retail services that a consumer can engage in when using their mobile device in brick-and-mortar stores. We developed and validated a nine-item, formative MIX index using survey data collected from fashion consumers in the United States (*n* = 1,267), United Kingdom (*n* = 370), Germany (*n* = 362), and France (*n* = 219). As measures of consumer engagement in omnichannel retailing using a mobile device, the index items with stronger factor loadings described in-store shopping activities whereas those with weaker factor loadings described activities related to behavioral targeting and social networking. These results suggest that retailers should give consumers the autonomy to independently find, evaluate and purchase merchandise in brick-and-mortar stores, thereby enabling them to co-create personalized shopping experiences as active participants within an omnichannel retail servicescape. Our findings also suggest that retailers should provide consumers with more authentic ways to build community and brand affiliations than mobile marketing and social media promotions.

Our validated MIX index is an effective measure for quantitatively gauging consumer engagement in omnichannel retailing. Academics could use the MIX index in conjunction with validated measures of consumer motivation for using mobile devices (Thakur, 2016) to discover new sources of value co-creation in retail stores, thereby bridging the need for research on enhancing consumer engagement in omnichannel retailing (Shankar et al., 2016). Practitioners could also use this set of validated survey questions to brainstorm, design, test, and iterate ways to increase consumer participation in their mobile in-store experience, providing a solid foundation for building successful omnichannel strategies (Vargo et al., 2008; Storbacka et al., 2016). As such, the MIX index could help retailers make more informed decisions about future investments and innovative strategies that tap into the consumer-centric and agile nature of omnichannel ecosystems.

## Theoretical Background

When engaging in a mobile in-store experience, an individual is not merely a buyer or shopper but becomes a participant within a sociotechnical system. They must counterbalance their online and offline mobile interactions, social encounters, product, and service experiences within a retail store. Hence, we used Activity Theory to develop contextually relevant and diverse MIX index items to capture the multifaceted shopping activities that could underscore a mobile in-store experience. Activity Theory (AT) is an interdisciplinary, theoretical approach to understanding the sociocultural and behavioral dimensions of human work originating from the fields of philosophy, cultural-historical psychology, and historical materialism (Leontev, 1974; Vygotsky, 1978; Engeström et al., 1999). This pragmatic and systematic philosophy for studying human behavior led AT to gain credence within human-computer interaction, where theories of cognition proliferated and once dominated the field (Nardi, 1996). AT became known as a powerful antidote to traditional, cognitive psychological theories within human-computer interaction (HCI), which traditionally favored laboratory experiments and often divorced technologies from their use contexts (Kuutti, 1995; Mickelsson, 2017; Kaptelinin and Nardi, 2018). As a result, AT provides a middle-ground framework that straddles theory and practice in order to help HCI researchers critically but pragmatically examine the ways end-users interact with the world through technologies (Kaptelinin and Nardi, 2012).

**Figure 1:**
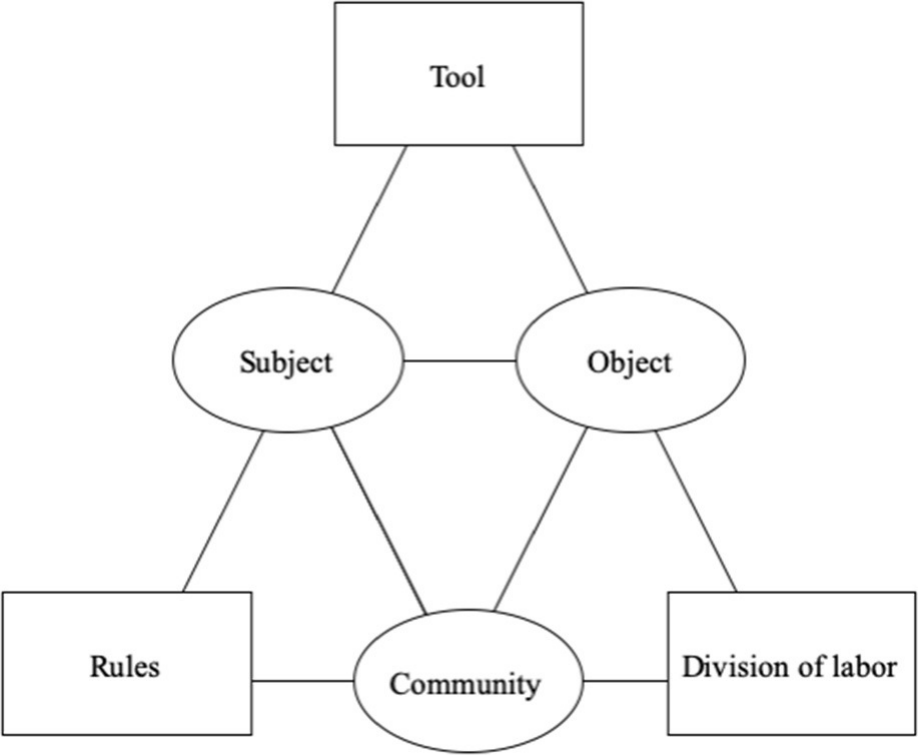
Activity Theory (AT) framework.

Activities are combinations of sensorimotor conditions and actionable goals that humans bring together in order to build purposeful experiences in society such as work, cultural, technical, or expressive practices. Activities are non-sequential and non-linear, and thus should be understood as constellations from which people can dynamically choose (Mickelsson, 2013). This thrownness inherent to the sensing and choosing of activities is what consumers and end-users interpret as experiences (Hepi et al., 2017). To that end, the relationship between a subject and some object or environment must provide a context for the activities that take place. More commonly in HCI and consumer research, a subject refers to a consumer or end-user whereas an object or environment refers to a home, workplace, or store. According to AT, a given tool or artifact such as an automobile, computer, or medical device mediates the subject–object relationship (see Figure 1).

Mediators (and acts of mediation) within HCI and media research capture the transformative qualities of computers and information technologies that enable subjects to influence objects, as well as objects to influence subjects through various activities (Bødker, 1989; Kuutti, 1995). From this perspective, mediation is not being used in the statistical sense of the word. AT, therefore, posits that activities are contingent upon three mediators stemming from the information systems and social science literature: community, rules, and division of labor (Engeström, 1987). A community is made up of people with shared interests in the same object, and within the AT framework, it mediates the subject–object (S–O) relationship. The rules are conventions and norms that mediate the subject–community (S–C) relationship because they build social connections within the community. The division of labor consists of the explicit and implicit structure of the community, which mediates the object–community (O–C) relationship since relevant social actors use a given tool or artifact determine how objects, such as retail stores, can be managed.

Within the HCI discipline, these acts of mediation are not intended to be prescriptive. As a meta-theory, the AT framework is more closely aligned with social constructivism than structuralism (Nardi, 1996). As a result, activities can be mixed according to the needs and desires of a subject (Mickelsson, 2013; Hepi et al., 2017). Due to this fluidity, mediators should not be called dimensions insomuch as micro-moments in which end-users or consumers participate in activities viscerally and construct their lived experiences. Along with this logic, a mobile in-store experience is comprised at the most basic level of activities that help consumers as subjects to interact through a given tool (mobile devices) with a focal object (the retail store) and community (social agents such as important others and retail workers).

We use this AT framework to develop our MIX index, which measures consumer engagement in mobile activities that enact the community, rules, and division of labor in physical retail stores. Therefore, our conceptualization of mobile in-store experiences is similar to what the marketing literature (cf. Lemon and Verhoef, 2016) refers to as “customer touch points” along the path-to-purchase process. Specifically, multichannel and service management research has analyzed these touch points to understand customers' options and decisions during their shopping journey, as well as drivers of their channel choice (e.g., Ansari et al., 2008; Bitner et al., 2008; Ko et al., 2009; Melis et al., 2015; Wang et al., 2015). In other words, the marketing literature emphasizes customers' thought processes over their actions in context, especially when it comes to measurement of customer experiences (Lemon and Verhoef, 2016; Verhoef, 2021). Our work follows more closely in the footsteps of a cadre of empirical studies that have adopted the AT framework to analyze customer experiences (i.e., Teixeira et al., 2012; Carlson et al., 2016; Mickelsson, 2017; Hsia et al., 2020). This latter research prioritizes the mediational role of tools or technologies between activities and broader domains of the human experience that transform how we communicate, learn, work, travel, shop, and play. In essence, AT is less concerned with cognition or *why* things are done and more concerned with action or *how* things get done. In contrast, marketing research has not measured customers' evaluation of interactive mobile activities within retail stores or with their community (Verhoef, 2021), which is why we ground the development of our MIX index in the AT framework.

## Development of the Mix Index

### Step-by-Step Procedure

To begin, we will define the conceptual domain of the MIX construct. The construct domain will be drawn from empirical findings within the field of omnichannel retailing. At the same time, we will show how current research intertwines with the AT framework and industry practices in an effort to build a construct that narrows the gap between theory and practice. Then, we will evaluate the potential dimensionality of the construct and generate a set of measurement items. Thereafter, we will discuss the data analytic procedures that will be employed to specify and purify the index. These steps are borrowed from the index construction process outlined in MacKenzie et al.'s (2005) *Journal of Applied Psychology* article on measurement models. At the same time, our paper intends to offer an initial validation of the MIX index and develop a set of measures that academic researchers or practitioners could use for academic field studies, store intercepts, or industry research. Due to the brevity of this study, we acknowledge that it is exploratory in nature and encourage future researchers to conduct additional testing of the MIX index. We discuss the limitations of this early research and describe avenues for future research in section Limitations and Future Research.

#### Defining the Construct Domain

First, the MIX construct should include community elements from the AT framework because academic researchers and industry experts have discovered that omnichannel retail strategies can depend on successful community-building. Traditionally, community in retailing distinctly referred to the cities, towns, and neighborhoods that surrounded stores and shopping districts. While these cultural geographies remain crucial for retail operations, stores in the 21st century are once again becoming communities in their own right (Alexander and Cano, 2020), and they are launching quirky pop-up shops (e.g., Wayfair.com), larger than life flagship stores (e.g., Burberry Regent Street), stores within stores (e.g., Story at Macy's), and showrooms (e.g., Everlane). By pairing contemporary retail formats with mobile in-store experiences, stores can welcome consumers into their real-time, brand communities through behavioral targeting. Behavioral targeting is defined as context-aware and real-time mobile services that offer product recommendations, attractiveness cues, and promotions gathered from in-store sensor data, user behavior, and purchase histories (Van der Heijden, 2006; Krumm, 2011). Previous studies have supported that consumers can derive value from behavioral targeting when the outcome is consistent with their shopping behaviors and goals (Blom et al., 2017). For example, Brynjolfsson et al. (2013) demonstrate that successful behavioral targeting helps consumers to produce value and seek social validation by giving them opportunities to curate content, seek feedback and increase their product knowledge.

Furthermore, consumers can socially transcend retail servicescapes by using their mobile devices to incorporate friends and family into their in-store shopping activities (Houliez, 2010). For example, they can take a photo of themselves wearing apparel and upload it to Facebook, Instagram, and Twitter in order to review and share product experiences within their social networks (Morris et al., 2014). In this way, mobile in-store activities can lead consumers to heighten their interactivity and sociability when shopping in retail stores, which enables the co-creation of value with retailers and social networks during each contextualized exchange (Gummerus and Pihlström, 2011; Ström et al., 2014). According to AT, this interactivity and sociability can represent the shared interests of consumers (subjects) within retail stores (objects). In effect, consumers are utilizing their mobile devices as tools to build senses of community by mediating the subject–object relationship.

Secondly, recent findings and industry practices suggest that the MIX construct should capture aspects of the rules domain from AT. Due to the transformative characteristics of mobile devices and the formation of global brand communities, retail markets are operating according to a new set of rules. Dart and Lewis (2017) describe a seismic shift within retailing wherein mobile devices yield consumers more negotiating power, empower them to seek greater transparency from retailers, and give them access to nearly limitless inventories and on-demand services. These new rules are embodied in consumer trends such as webrooming and showrooming, which involves the assessment of products online prior to visiting a physical store and the purchasing products online from a competing retailer during or after a store visit (Jing, 2018).

To address these challenges, retailers must offer flexibility and selectively grant consumers permission through mobile devices to defy some conventional social norms within retail stores such as standing rather than skipping the checkout line, adhering to rigid store policies for couponing, and waiting for new products to arrive at the store (Shi et al., 2020). For example, Best Buy and Lowe's have invested significantly in mobile apps and technologies that tap into new, self-service opportunities presented by mobile media and devices (Pearson, 2017). Recent studies also demonstrate that mobile, self-service technologies in retail stores produce customer satisfaction, reduce wait-time perceptions, and create perceived value (Inman and Nikolova, 2017; Djelassi et al., 2018). Therefore, consumers are leveraging their mobile devices to mediate the subject–community relationship. Consumers (subjects) are effectively creating a new normal wherein mobile devices lead them to renegotiate their interactions with social agents such as important others and retail workers (community).

Thirdly, the MIX construct must include aspects of the division of labor domain from AT to mirror the new forms of consumer engagement in the omnichannel environment. Current research and industry practices illustrate that mobile devices have shifted the division of labor between frontline retail associates and store visitors. Traditionally, a salesperson or end-provider was the locus of activity and value creation within retail stores. Over the past decade, mobile devices and accelerating consumer demands have democratized store environments and increased consumers' direct involvement with the selling of goods and services (Lewis and Dart, 2014). RFID and Bluetooth connections especially have led consumers to increasingly assume some responsibilities from sales associates through mobile apps, such as wayfinding (e.g., REI), gathering product information (e.g., Sephora), and building gift lists (e.g. Target). Amazon Go, perhaps, is at the extreme end of these mobile in-store activities because consumers may optionally choose to never interact with sales associates (Grewal et al., 2017). Additionally, findings suggest that such participatory behaviors can strengthen customer relationships, enhance customer loyalty, and increase hedonic purchases (Juaneda-Ayensa et al., 2016; Mustak et al., 2016; So et al., 2016; Huang and Yang, 2018). Likewise, consumer participation is indicative of a gradual power-shift in which mobile devices are enabling consumers to collaborate with social agents (community) to manage and navigate retail stores (objects) in new ways. Specifically, consumers can use mobile devices to mediate the object–community relationship, between physical stores and social agents within retail servicescapes.

#### Evaluating the Conceptual Dimensionality

In the former sections (Development of the MIX Index and Step-by-Step Procedure), we defined the construct domain and postulated that three types of mobile-mediated activities can represent the MIX construct: community, rules, and division of labor. This observation was drawn from AT and substantiated with current m-commerce and omnichannel research (e.g., Grewal et al., 2017; Inman and Nikolova, 2017; Alexander and Cano, 2020; Shi et al., 2020). The next step calls for additional theoretical support to ascertain the dimensionality of the construct (MacKenzie et al., 2005). According to Jarvis et al. (2003), conceptual dimensionality means that a researcher must heed attention to the levels of abstraction within a construct. Indeed, the AT framework and literature support the notion that consumers and retailers create a mobile in-store experience from three types of activities. Nevertheless, the MIX construct should be unidimensional in nature, rather than having three unique subdimensions that give rise to a mobile in-store experience since activities and mediators are fluid.

AT asserts that, even though technological mediation appears to crosscut three domains, activities do not occur within a specific order and can be mixed or matched according to the needs and desires of the subject to produce an experience (Mickelsson, 2013; Hepi et al., 2017). This subjectivity and spillover are central tenets of the AT framework. By creating subdimensions, we would violate this basic principle and suggest that mobile-mediated activities fall into neat boxes. This index will represent a single trait: intentions to engage in omnichannel retailing through a mobile device. Morwitz and Munz (2021) define *intentions* as how much a consumer resolves or desires to act in a certain manner. As such, the unidimensional MIX construct is similar to other global scales that tap into behavioral and usage intentions including validated measures of prosocial behavior (Baumsteiger and Siegel, 2019), desires for new products (Lynn and Harris, 1997), social media use (Chintalapati and Daruri, 2017), online innovativeness (Goldsmith, 2001), and digital coupon redemption (Nayal and Pandey, 2020).

#### Generating a Set of Measurement Items

Mobile devices that consumers currently use in retail stores, such as tablets and smartphones, are similarly transformative as the tools described in the AT framework. According to Shankar et al. (2010), the key properties of mobile devices in the omnichannel retail environment include capabilities and qualities such as ultra-portability, location sensitivity, untetheredness, and personalization. These sociocultural and technical characteristics have led consumers to integrate mobile devices into their shopping trips long before mobile apps and social media were mainstream (Rigby, 2011). Likewise, retailers have to consider how they might leverage this mobile consumer behavior to produce commercial opportunities (Shankar et al., 2010; Dart and Lewis, 2017). A mobile in-store experience, therefore, includes shopping activities and retail services that are initiated by consumers and retailers within an omnichannel retailing environment.

Hence, we iteratively developed nine interaction intentions to capture elements of consumer engagement in omnichannel retailing through a mobile device by considering the major AT domains (community, rules, and division labor) and key properties of mobile devices (ultra-portability, location sensitivity, untetheredness, and personalization).

Based on this information, we developed three interaction intentions related to the notion of community during consumer engagement in omnichannel retailing. These activities are consistent with behavioral targeting and the personalization properties of mobile devices, which consumers can harness through affiliation with social networks and brands in order to enhance their immersion in retail servicescapes:

- “I would use my mobile device in-store to identify myself as a loyal customer so I could get personalized offers.”
- “I would use my mobile device in-store to receive product recommendations based on my purchase history.”
- “I would use my mobile device in-store to review products by uploading photos to social media.”

Based on these research insights, we developed three interaction intentions related to rules during consumer engagement in omnichannel retailing. These in-store shopping activities leverage the untetheredness and ultra-portability properties of mobile devices, which consumers can use to circumvent old rules and practice new rules during their interactions with relevant community members such as salespeople, shoppers, and important others:

- “I would use my mobile device in-store to review special coupons/promotions sent to my email or phone account.”
- “I would use my mobile device in-store to order out-of-stock products if they shipped to my home for free.”
- “I would use my mobile device in-store to pay for the products I want to buy if I could then skip the check-out line.”

As a result, we developed three interaction intentions pertaining to the division of labor during consumer engagement in omnichannel retaining. These in-store shopping activities build on the location sensitivity and untetheredness properties of mobile devices, which enable consumers to assume some employee responsibilities and co-create value within retail servicescapes, thereby impacting the division of labor as denizens in stores:

- “I would use my mobile device in-store to identify my location on a store map so I could find products.”
- “I would use my mobile device in-store to obtain product information and reviews by scanning labels/tags.”
- “I would use my mobile device in-store to build a gift/shopping list by scanning labels/tags.”

After generating the interaction intentions, we aimed to assess the content validity of the MIX index. To establish content validity, the full domain of the MIX construct should address the different ways that consumers can engage in omnichannel retailing through their mobile devices. Yet, mobile-mediated activities depend on the technological capabilities that software companies and retailers have hardwired into retail stores such as WiFi, Bluetooth, and RFID sensors. As a result, the MIX construct must be pragmatic and reflect the scope of mobile-mediated activities that are conceivable and available within a large number of brick-and-mortar stores.

To address these constraints, we conducted a semi-structured interview with the Vice President of Demandware (now Salesforce Commerce Cloud) who was the financial sponsor of this research. Demandware's cloud-based software supported retail operations for 350 well-known stores and 1,600 online stores worldwide, thus this executive was knowledgeable about the omnichannel capabilities that most retailers enable consumers to access while shopping. During the interview, this executive confirmed that the items described as mobile in-store shopping activities and retail services were currently supported by many department stores and specialty retailers. Thereafter, we pre-tested the index items with 10 undergraduate students enrolled in an upper-level survey and research methodology course for comprehension.

#### Determining the Relationship Between the Construct and Its Measures

After generating the measurement items, we carefully assessed the relationship between the construct and its measures to prepare for data collection and scale purification. The rationale for taking this step is that many researchers underestimate the importance of choosing between a formative or reflective construct. Data simulations prove that when a researcher misspecifies the construct and its relationship with the measures, it leads to biased model estimates and inaccurate hypothesis testing (Jarvis et al., 2003). Within a *formative* construct, causality flows from the indicators to the latent variable in contrast to a *reflective* construct, in which the latent variable is hypothesized to impact or cause measured behaviors. Additionally, the indicators that underlie a formative construct are not presumed to be interchangeable or internally consistent. The indicators do not produce equivalent changes in the latent variable because they may represent a composite of mutually exclusive feelings, attitudes, or behaviors (Jarvis et al., 2003). Hence, the MIX index should be represented as a formative construct. Because the MIX index items are conceptualized as interaction intentions, these indicators are hypothesized to independently and cumulatively impact the latent variable.

### Study 1

#### Data Collection and Sample Characteristics

To collect data for purifying the nine-item MIX index, we contracted with SurveyMonkey Audience to recruit consumers who had recently purchased fashion products and administer to them our online survey. Fashion products were defined as, “Items that you can wear such as clothing, shoes, and accessories, i.e., belts, ties, hats, scarves, leather goods, jewelry, etc.” We limited our survey to this context because fashion retailers have been recognized as innovators in the m-commerce and omnichannel space (Röcker, 2009; Hansen and Sia, 2015). Furthermore, fashion consumers interact heavily with products and salespeople in-store given the sensorial and symbolic aspects of fashion goods (Küchler and Miller, 2005). As a pre-screening question, respondents were asked, “How many fashion products did you purchase in the past 3 months?” Respondents were disqualified from participation if they purchased less than one fashion product in the past 3 months.

It was also imperative to recruit consumers with prior m-commerce and omnichannel shopping experience so that they could understand and respond accurately to the mobile in-store activities that would be presented to them in the survey (Hallikainen et al., 2019; Morwitz and Munz, 2021). Therefore, we pre-screened survey respondents based by asking them, “What technology do you use when shopping for fashion products?” Respondents were screened out if they did not choose 4 (almost always) or 5 (always) for either the “mobile tablet” or “mobile phone” options. After the pre-screening, 1,267 respondents from the United States and 951 respondents from Western Europe participated in the online survey (see Table 1), which constituted a 49.8% average response rate. When presented with the MIX index items, respondents were specifically directed to “Read the following statements about in-store mobile shopping services offered by fashion retailers and select an appropriate response.”

**Table 1:** Study samples.

	**Study 1 (*n* = 1,267)**	**Study 2 (*n* = 951)**
**Gender**		
Male	47.4%	44.9%
Female	51.7%	54.6%
Decline to answer	0.9%	0.5%
**Age**		
Under 24	10.4%	12.9%
25–34	43.2%	29.8%
35–44	26.2%	29.3%
45–54	12.4%	18.4%
55–64	4.7%	7.2%
≥55	2.8%	2.4%
Decline to answer	0.3%	0%
Household income^*^		
<29,000	17.8%	35.3%
30,000–44,999	15.3%	30.6%
45,000–59,999	12.6%	14.9%
60,000–74,999	13.4%	9.3%
75,000–99,000	14.6%	5.7%
≥100,000	25.3%	3.5%
Decline to answer	1.0%	0.7%
**Citizenship**		
USA	100.0%	0%
United Kingdom	0%	38.9%
Germany	0%	38.1%
France	0%	23.0%

* *Non-equivalent: USA ($), UK (£), Germany, and France (€)*.

#### Model Specification and Reliability Assessment

Mplus 7.4, a structural equation model (SEM) program, was used to specify and evaluate a formative measurement model (Diamantopoulos and Winklhofer, 2001). After specifying that the hypothesized nine-item MIX index was a formative construct, a Confirmatory Factor Analysis (CFA) was performed to test the reliability and validity of this index (Kline, 2011). After building the MIMIC model, the initial model fit was good (see Table 2): *X*^2^ (6) = 27.79, RMSEA = 0.06, CFI = 0.99, TLI = 0.97, SRMR = 0.01 (Hu and Bentler, 1999; Chen et al., 2008). Next, the reliability of the nine-item MIX index was examined at the indicator level rather than at the construct level. The reliability cannot be assessed at the construct level because formative indicators are not presumed to be correlated (MacKenzie et al., 2011). As a result, interconstruct correlations are not substantively meaningful for a formative construct. The indicator reliabilities, alternatively, can be evaluated by looking at the *Z*-scores for the individual factor loadings (MacKenzie et al., 2011). The *Z*-scores for the formative indicators were >1.96, which suggested that the MIX index was reliable at the indicator level. A multiple regression equation generated the factor loadings, however, which increases the very minor risk of multicollinearity in a formative model. Multicollinearity is a sign of redundant indicators and prevents the researcher from effectively capturing the essential domains of a construct (Grewal et al., 2004; Dickinger and Stangl, 2013). To test for multicollinearity, bivariate correlations were calculated between the index items. The results showed that two of the items had correlations >0.70. Therefore, we individually regressed the problematic items onto the remaining seven items. The Variance Inflation Factor (VIF) for each regression statistic (<1.0) was less than the recommended 3.0 cutoff, which indicated multicollinearity was not present (Petter et al., 2007).

**Table 2:** Model specification and cross-validation.

	**Study 1 (USA:** ***n*** **=** **1,267)**			**Study 2 (Europe:** ***n*** **=** **951)**		
	**Std. (γ)**	***Z*-score**	**Mean**	**Std. (γ)**	***Z*-score**	**Mean**
**Stronger factors**						
…to review special coupons/promotions sent to my email or phone account.	0.17	6.72^***^	4.87	0.22	10.80^***^	4.24
…to identify my location on a store map so I could find products.	0.16	6.41^***^	4.09	0.22	9.12^***^	3.57
…to obtain product information and reviews by scanning labels/tags.	0.22	7.95^***^	4.38	0.26	9.40^***^	3.90
…to build a gift/shopping list by scanning labels/tags.	0.29	11.42^***^	4.20	0.27	10.46^***^	3.68
…to order out-of-stock products if they shipped to my home for free.	0.83	82.77^***^	4.53	0.85	82.97^***^	4.12
…to pay for the products I want to buy if I could then skip the check-out line.	0.80	73.69^***^	4.25	0.77	65.81^***^	3.83
**Weaker factors**						
…to identify myself as a loyal customer so I could get personalized offers.	0.09	3.78^***^	4.53	0.04	1.85	3.94
…to receive product recommendations based on my purchase history.	0.06	2.18^*^	4.17	0.04	1.71	3.60
…to review products by uploading photos to social media.	0.07	3.08^**^	3.47	0.02	0.16	3.02

*** p < 0.001,

** *p < 0.05*,

* *p < 0.10*.

#### Convergent and Discriminant Validity

Next, we assessed the convergent validity of the MIX index and considered the extent to which its items account for variance in the latent (MIX) construct. The convergent validity of the survey items was assessed with the error term of the latent construct (Williams et al., 2003). The rationale is that the error term should be <0.50 assuming the items fully capture the construct meaning. The error term for the latent construct was 0.29, which confirmed the convergent validity for the nine-item MIX index. We also assessed the discriminant validity of the MIX index. Discriminant validity is the ability to distinguish a construct and its indicators from other constructs. In order to test the discriminant validity of the MIX index, it was critical to demonstrate that the construct was unique from similar forms of mobile use and communication. To that end, a measure of electronic word-of-mouth (eWOM) was added to the confirmatory factor analysis. According to recent studies, eWOM occurs in an omnichannel setting when consumers share and review opinions about products sold in physical stores through websites and mobile devices (Flavián et al., 2020; Grewal et al., 2020; Tyrväinen et al., 2020). Thus, it was important to distinguish this closely related but different phenomenon from the activities that comprise a mobile in-store experience.

eWOM was measured with the following question: “How do you tell others whether you like/dislike fashion products after you buy them?” Respondents were given six choices (text, email, tweet, blog, Facebook, or Pinterest), and they were asked to rate their eWOM frequency on a five-point, Likert scale (never to always). In the model, we allowed the MIX index items and eWOM to be freely correlated: *X*^2^ (70) = 1991.38, RMSEA = 0.12, CFI = 0.86, TLI = 0.83, SRMR = 0.26. The latent covariance between the MIX index and eWOM items were then constrained to 1.0: *X*^2^ (71) = 3017.23, RMSEA = 0.14, CFI = 0.78, TLI = 0.74, SRMR = 0.27. By performing a chi-square difference test, it is possible to see whether the constraint improves the model fit. In other words, if the unconstrained model fits the data significantly better than the constrained model with the latent constructs perfectly correlated, then discriminant validity exists (MacKenzie et al., 2011). After doing so, the chi-square difference test proved to be significant: *X*^2^ (1) = 1025.85, *p* < 0. 001. Therefore, the MIX index had discriminant validity.

### Study 2

#### Data Collection and Sample Characteristics

In order to further test the nine-item MIX index, survey data was collected from consumers in Western Europe 2 weeks later and retested the formative measurement model. Cross-validation can help establish whether sample characteristics have any effect on psychometric properties and determine if a scale is reliable within different populations (Churchill and Peter, 1984). Prior to commencing the online study in Western Europe, professional survey linguists translated and back-translated it from American English into British English, German, and French. Once again using SurveyMonkey Audience panels, online survey data was collected from respondents in the United Kingdom (*n* = 370), Germany (*n* = 362), and France (*n* = 219) (see Table 1). These countries collectively represent the largest share of the m-commerce market in Western Europe (Enberg, 2018). This second study, therefore, provided a rigorous test of the MIX index's reliability and validity.

#### Model Specification and Reliability Assessment

Using Mplus 7.4 once again and drawing upon survey data from these European samples, the nine-item MIX index was placed into a Multiple Indicators Multiple Causes (MIMIC) model (Jöreskog and Goldberger, 1975). In order to replicate the prior study, we defined the first seven items from the index as formative while emitting paths to the remaining two items (Jarvis et al., 2003). After doing so, the initial model fit was good (see Table 2): *X*^2^ (6) = 10.73, RMSEA = 0.03, CFI = 0.99, TLI = 0.99, SRMR = 0.01 (Hu and Bentler, 1999; Chen et al., 2008). Next, we examined reliability at the indicator level. The *Z*-scores for three of the nine items were <1.96. Upon reviewing the item factor loadings from Study 1, we found these same items previously had the weakest factor loadings and *Z*-scores. Their inability to reach significance when retesting the MIX index confirmed that these items were weaker than the others. Dropping an item from a reflective model does not alter the meaning of the construct. However, dropping an item from a formative model eliminates an essential piece of the construct, and by extension, necessitates strong theoretical justification (Diamantopoulos et al., 2008; Edwards, 2011). For these reasons, the weaker items were not dropped from the index. After testing the reliability of the items, VIF scores were calculated to assess multicollinearity. None of the VIF scores exceeded the 3.0 cutoff, which confirmed that multicollinearity was not an issue.

#### Convergent and Discriminant Validity

According to prior research (Williams et al., 2003), an error term <0.50 conveys an acceptable level of convergent validity. In Study 2, the error term for the latent construct was 0.33, supporting that the MIX index had convergent validity. In order to test its discriminant validity, we once again ran a confirmatory factor analysis and allowed the nine-item MIX index and the frequency measure of eWOM to be freely correlated: *X*^2^ (70) = 1723.89, RMSEA = 0.11, CFI = 0.86, TLI = 0.83, SRMR = 0. 24. Subsequently, we constrained the latent covariance between both measures to 1.0: *X*^2^ (71) = 2620.66, RMSEA = 0.14, CFI = 0.78, TLI = 0.74, SRMR = 0.26. The chi-square difference test proved to be significant: *X*^2^ (1) = 896.77, *p* < 0. 001. Therefore, the nine-item MIX index had discriminant validity.

## Discussion and Implications

Drawing on insights from Activity Theory (AT), we developed and validated a new formative measure of consumers' intended use of a mobile device to engage in activities and services when shopping in retail stores. The nine items in this mobile in-store experience (MIX) index describe an omnichannel environment wherein consumers can co-create value by leveraging the key properties of their mobile devices, namely ultra-portability, location sensitivity, untetheredness, and personalization. Consistent with AT, the MIX index validates that the sociocultural and technical qualities of mobile devices enable consumers to transcend the retail store environment through a nexus of activities. These omnichannel activities afforded by mobile devices are discontinuous, yet they are synthesized through community, rules and division of labor.

The six MIX index items that were strong indicators of consumer engagement in omnichannel retailing measured whether they would use a mobile device to enact the following in-store shopping activities:

1. Review special coupons/promotions sent to their email or phone account.
2. Identify their location on a store map in order to find products.
3. Obtain product information and reviews by scanning labels/tags.
4. Build a gift/shopping list by scanning labels/tags.
5. Order out-of-stock products that are shipped to their home for free.
6. Pay for products in order to skip the checkout line.

Within this described retail servicescape, consumers do not need to rely on a salesperson and be subjected to undesirable sales pressure when browsing merchandise in-store. They can instead enjoy an untethered shopping experience delivered to them via their mobile devices. Therefore, our results suggest that retailers should identify and give consumers additional opportunities for autonomy through use of their mobile devices in stores, building upon the rules and division of labor domains of the AT framework.

The three MIX index items that were weak indicators of consumer engagement in omnichannel retailing measured whether they would use a mobile device to participate in the following behavioral targeting and social networking activities:

1. Identify themselves as loyal customers to received personalized offers.
2. Receive product recommendations based on their purchase history.
3. Review products by uploading photos to social media.

Without a need to escape crowds and associated negative emotions by engaging with behavioral targeting (Andrews et al., 2016), our findings suggest that fashion consumers in the United States and Western Europe are more engaged and empowered by shopping activities and retails services that give them the freedom to independently evaluate merchandise using their mobile devices in retail stores. Therefore, our results call into question prior research on the benefits of behavioral targeting (Brynjolfsson et al., 2013; Blom et al., 2017), especially within an omnichannel retail servicescape. Our results are consistent with prior research on social networking (Inman and Nikolova, 2017), which suggests that consumers have privacy concerns about using mobile devices in-store for promotional activities. Given that behavioral targeting and social networking activities fit within the community domain of the AT framework, our results suggest that retailers should provide consumers with more authentic ways to build community and brand affiliations than mobile marketing and social media promotions. For example, Högberg et al. (2019) found that consumers responded favorably to behavioral targeting when gamification was added to a mobile in-store experience and aligned with their shopping tasks. An industry example would be Gucci's “Places” Project. This mobile game encouraged consumers to visit special locations and collect virtual badges – before, during, and after store visits – that melded with the cultural and aesthetical histories behind their current product lines (Salibian, 2017).

### Limitations and Future Research

Despite the significance and veracity of our findings, there are some potential limitations of the current research, which could be improved upon through future replication or extension. Any study that uses a common method as the primary tool for data collection may be at risk of common method variance, which is a form of systematic error variance that stems from measuring the constructs with a single method (Podsakoff et al., 2003). We used an online survey in both studies to collect data and develop the nine-item MIX index. Hence, common method variance could impact the validity and interpretation of our measures. That being said, we incorporated many of the recommended tools for controlling common method variance, such as the counterbalancing of question order, ensuring and protecting respondent anonymity and gathering data from multiple samples. By validating the MIX index on actual behaviors, the risk of common method variance could be reduced. For example, researchers could observe consumers' use of mobile devices in retail stores, compare these behaviors with the measured omnichannel shopping activities, and then interview them about it.

Another possible limitation of our research is the content validity of the nine-item MIX index. The goal of this study was to develop a short index for academic field studies, store intercepts, and industry research. Also, the MIX index is a pragmatic scale that reflects the mobile technologies currently available within the omnichannel environment on a wide scale rather than taking cues from niche markets or offering a snapshot of the future. Thus, we consulted a significant body of literature and generated the measurement items with theoretical support from human-computer interaction. Nevertheless, the qualitative phase of this research included an interview with one industry expert, which may have limited our ability to explore the full domain of the MIX construct. Future empirical studies should consider using larger expert panels and contemporary Q-sorting to conduct a deeper assessment of content validity (Petter et al., 2007).

In a similar vein, we encourage researchers to expand the nine-item MIX index as omnichannel retailing capabilities evolve with innovations including the Internet of Things, augmented/virtual reality, and social media (Grewal et al., 2020). Equally, it would be valuable to examine aspects of store design (Alexander and Cano, 2020), atmospherics (Bitner, 1992; Rosenbaum and Massiah, 2011), and the customer journey (Grewal and Roggeveen, 2020). The inclusion of emerging technologies and customer experience variables will provide rich opportunities for adding the MIX index to predictive and cognitive models of technology acceptance. For example, the MIX index could be modeled as an outcome variable in the extended Unified Theory of Acceptance and Use of Technology model (UTAUA2; Venkatesh et al., 2012). By regressing the MIX index onto the UTAUA2 antecedents (i.e., hedonic motivation, social influence, and price value), future research can investigate why and whether consumers have preferences for different omnichannel activities that constitute a mobile in-store experience.

Moreover, the MIX index should be tested within diverse cross-cultural contexts. For example, the index items that capture behavioral targeting and social networking activities were found to be the weakest during our cross-validation of the MIX index, but this weakness was more pronounced in Western Europe than the United States. These subtle differences may be related to cultural variation. Hence, future researchers should probe whether cultural variation and related attitudes toward mobile technology and shopping are driving these subtle differences by collecting data from western and non-western cultures. Lastly, given the moderating effects of gender on fashion retailing within cross-cultural contexts (Nysveen et al., 2005), researchers should evaluate the MIX index within other industries, such as food services, hospitality, automotive, banking, and consumer technology (Ström et al., 2014).

## Conclusion

This research suggests that an engaging mobile in-store experience should be an amalgam of physical and digital activities that produce a seamless shopping journey. In-store activities should not simply be a migration of pre-existing e-commerce capabilities onto mobile devices. Instead, they should give consumers the autonomy to independently find, evaluate and purchase merchandise in brick-and-mortar stores, thereby enabling them to co-create personalized shopping experiences as active participants within an omnichannel retail servicescape. This conceptualization of a mobile in-store experience is consistent with Activity Theory (Kaptelinin and Nardi, 2012), as well as the literature on consumer co-creation (Prahalad and Ramaswamy, 2004; Edvardsson et al., 2010; Kohler et al., 2011), which similarly emphasizes the participatory and consumer-centric nature of contemporary marketing practices and technology design. Retail executives can use the validated MIX index to prepare strategic investments in mobile technology applications and capabilities for retail stores within their omnichannel operations. The nine-item MIX index is also well-suited for consumer surveys, which also makes it an attractive measure of consumer engagement in omnichannel retailing for future academic research.

## Data Availability Statement

The original contributions presented in the study are included in the article/supplementary material, further inquiries can be directed to the corresponding author.

## Ethics Statement

The studies involving human participants were reviewed and approved by Human Subjects Protection Program, University of Arizona. The patients/participants provided their written informed consent to participate in this study.

## Author Contributions

AB designed the research study and was responsible for overall project administration. CL analyzed the survey data and drafted an initial manuscript. All authors contributed to survey design, manuscript revisions, and approved the submitted version.

## Conflict of Interest

CL and AB consulted with Demandware (now Salesforce Commerce Cloud) to ensure that MIX index items accurately described shopping activities and retail services available to consumers using their mobile device in brick-and-mortar stores. Demandware was not involved, however, with any aspect of this publication, including the analysis and interpretation of reported survey data.
